# Opinion: a call for safe therapeutic and gastric interventions for adolescents and young adults with obesity and transplant-indicated leukemia

**DOI:** 10.3389/fonc.2026.1813561

**Published:** 2026-05-07

**Authors:** Miriam B. Garcia, Paul Perales, David Santos, Ching-Wei D. Tzeng, Branko Cuglievan, Irtiza N. Sheikh

**Affiliations:** 1 Department of Pediatrics, The University of Texas MD Anderson Cancer Center, Houston, TX, United States; 2 Miami Cancer Research Center, Miami, FL, United States; 3 Department of Surgical Oncology, The University of Texas MD Anderson Cancer Center, Houston, TX, United States

**Keywords:** AYA, GLP-1RA, HSCT, leukemia, minimally invasive gastric surgery, obesity

## Introduction

In 2019, Transplantation and Cellular Therapy published a manuscript from Doney et al. titled “Impact of body mass index (BMI) on outcomes of hematopoietic stem cell transplantation (HSCT) in adults” where obesity was shown to lead to an increase in non-relapse mortality (NRM) in adult patients (1). Multiple studies have similarly demonstrated the risk obesity poses to survival in HSCT; however, there is little pediatric or adolescent/young adult (AYA) (ages 15–39 years) literature proposing surgical or medical management of obesity in patients undergoing active leukemia therapy, much less HSCT. In 2025, Frontiers in Oncology published “A deep dive into the surgical and pharmacological considerations of obesity in chronic myeloid leukemia (CML): a narrative review” where Ghasoub et al. presented the possibility of impaired drug absorption in patients with CML and history of previous gastric bypass (2). While surgical and medical management of obesity such as gastric bypass or other minimally invasive interventions such as gastric balloon or plication may come with some risk for patients with leukemia, they may also provide a path to a HSCT, which remains the only curative option for certain patients with poorly prognostic leukemia. Current studies are centered on adult patients with leukemia; however, there is a need to adapt and refine interventions for pediatric and AYA populations. AYA patients with leukemia already face unique age-related prognostic challenges; hence, a comprehensive approach is needed to address supportive options—pharmacological and surgical—for AYAs with coexisting obesity and leukemia who require HSCT.

## Background

Adolescents and young adults with obesity who are diagnosed with leukemia are more vulnerable to treatment complications, greater morbidity, and poorer survival (2). Although limited, data also suggest that obesity negatively impacts outcomes in AYAs undergoing allogeneic HSCT related to an association between higher BMI and worse survival (3).

### Obesity in AYA patients with acute leukemia

Obesity in AYAs contributes to worse toxicity and poorer outcomes in the treatment of leukemia (4–6). AYA patients who are overweight or obese with acute lymphoblastic leukemia (ALL) exhibited higher incidence of grade 3/4 hepatotoxicity and hyperglycemia compared to their non-overweight counterparts (60.7% vs. 42.2%, *p* = 0.0005, and 36.4% vs. 24.4%, *p* = 0.014, respectively) (4). Additional risks attributed to obesity include hyperbilirubinemia, thrombosis, and anaphylaxis, which can limit chemotherapy dosing to mitigate further toxicity (7, 8). In turn, dose reductions, potential omission of planned chemotherapy, and possible alterations in the pharmacokinetics of chemotherapy may play a role in poorer prognosis of AYA patients with ALL who suffer from obesity (7, 9).

Data from 388 AYA patients undergoing treatment with Dana Farber Consortium pediatric regimens for ALL between 2008–2012 demonstrated that 46.6% of patients were categorized overweight or obese and experienced higher rates of non-relapse mortality (11.7 vs 2.8%), lower EFS (63% vs 77% at 4 years), and poorer OS (64% vs 83%, *p* = 0.0001). This data suggests a correlation between obesity and overweight status and an increased risk for positive end of induction measurable residual disease (10).

### Impact of obesity on outcomes in Pediatric and AYA patients following HSCT

While there is limited data on the impact of obesity in AYAs undergoing HSCT, obesity in this subset of patients can negatively influence outcomes and is a factor to consider prior to transplantation. For instance, in pediatric and AYA patients undergoing HSCT for hematological malignancies, higher BMI has been associated with higher transplant-related mortality (TRM) (11). However, conclusions on the impact of obesity on HSCT outcomes in this population are difficult to reach due to the limited data. As such, a literature search was performed in search of similar studies in hematologic conditions requiring HSCT and revealed a review of pediatric and adolescent patients undergoing HSCT for aplastic anemia where obesity was predictive of survival outcomes, with patients considered overweight having significantly lower overall survival (OS) rates compared to those with lower BMIs (12). Notably, conclusions regarding outcomes should be made cautiously as conditions for patients undergoing HSCT for malignant conditions are different from that of aplastic anemia, i.e. different conditioning chemotherapies, graft-versus-host disease (GVHD) prophylaxis drugs, and distinct pathophysiologies.

### Practice changes for HSCT patients with obesity

Keeping these studies in mind, the risk of TRM secondary to obesity has led to changes in practice at the Children’s Cancer Hospital of MD Anderson Cancer Center. In order to avoid increased drug adverse effects due to high weight-based dosing, our practice includes normalizing the dosing of conditioning chemotherapy such as cyclophosphamide or fludarabine to use ideal body weight in patients who are overweight or obese, use of time-sequential busulfan with pharmacokinetics, use of prophylactic defibrotide to mitigate the risk of sinusoidal obstructive syndrome (SOS), as well as aggressive use of continuous renal replacement therapy. Despite these measures, toxicities and limitations related to obesity emerge, including decreased ambulation, fluid overload secondary to high maintenance intravenous fluids rates which further worsens the risk for SOS, and in some instances, death due to end organ damage.

Our approach to decrease the impact of obesity on transplant-related outcomes is reinforced by data that demonstrates that patients with obesity undergoing HSCT are subjected to an increase in NRM. Use of ideal body weight for certain patients rather than absolute body weight for the dosing of conditioning chemotherapy is supported by Doney et al. They demonstrated that obesity increased the incidence of NRM with higher conditioning regimen intensity, particularly in patients receiving myeloablative conditioning with total body irradiation (TBI) (1). Moreover, prior to HSCT, we use the Hematopoietic Cell Transplantation-specific Comorbidity Index (HCT-CI) to identify relevant comorbidities, including obesity, to support risk assessment in HSCT candidates (13). In addition to assessing the impact of obesity on morbidity risk, this tool allows us to evaluate organ vulnerability, including susceptibility to injury from factors such as high-dose conditioning regimens (14).

While there are a small number of large AYA-centered studies that evaluate the impact of obesity on HSCT outcomes, the current literature that exists is consistent with findings in adult literature. For instance, one study found 3-year NRM at 33% in patients with obesity vs. 19% in patients with normal-weight (15). GVHD, transplant-associated thrombotic microangiopathy (TA-TMA), and infections were major contributors to the increased NRM. Correspondingly, in a single center pediatric study that evaluated the impact of pre-transplant BMI on HSCT outcomes, higher BMI led to a significantly higher risk of acute GVHD and another study found a higher risk of TA-TMA in such patients (16, 17). These adverse effects may also be related to the contribution of obesity-related glucose intolerance to higher infection risk and a pro-inflammatory state increasing endothelial injury risk, as seen with higher TA-TMA incidence in patients ≥85th percentile for BMI (18). Similar findings were observed in Doney et al.’s work and other adult HSCT populations, including an elevated risk of SOS and liver dysfunction (1, 18). To improve the prevention and management of obesity-related co-morbidities surrounding HSCT, it is crucial to determine if these findings are similar in AYA populations.

## Discussion

Obesity remains a critical problem for the HSCT community, particularly in AYAs; however, uniform guidance does not exist on the optimal timing for transplantation in these patients with obesity. Moreover, alteration in factors such as the dosing of conditioning regimen chemotherapy, post-transplant immunosuppression, as well as antimicrobials, impact outcomes following transplantation due to suboptimal dosing secondary to high BMI (19–21). Data specific to AYAs has not yet been developed and so we extrapolate our discussion from adult studies to fill the evidence gap in pediatric and adolescents. For these reasons, we strongly urge for the development of guidelines on treatment options that directly address how to manage and mitigate the downstream effects of obesity in the pre-transplant and transplantation period in AYA patients who undergo HSCT for high-risk acute leukemia.

### Impact of high BMI on outcomes following HSCT

High BMI may be associated with worse outcomes in AYA HSCT due to endothelial injury considering that obesity is associated with a pro-inflammatory state. This is underlined by the higher incidence of TA-TMA, especially in those with BMI in the ≥85^th^ percentile in patients <20 years old (17). Similarly, an adult study showed patients who are obese or overweight undergoing HSCT had a higher incidence of HSCT-related complications such as SOS and liver dysfunction. This demonstrates the strong correlation between obesity and preventable HSCT complications. It also highlights the need for further studies to assess the impact of obesity on organ function during AYA HSCT, as well as interventions that mitigate the development of major contributors to NRM through weight management (18).

### Strategies to mitigate obesity-related toxicities in HSCT

In this commentary, we prioritize a call for minimally invasive options and strategies to mitigate toxicities attributed to obesity. This approach may avoid significant surgery in a classically immunosuppressed group as well as delays in leukemia treatment or HSCT due to wound healing.

#### Glucagon-like peptide receptor agonists

Glucagon-like peptide receptor agonists (GLP-1RA) (e.g. liraglutide, semaglutide) are currently approved for patients ≥12 years and reportedly can induce 16.1% weight loss over 68 weeks in adolescents (22). These agents may play a role in BMI reduction in lieu of surgical options prior to the initiation of conditioning chemotherapy. Considering that there is little reliable data on the use of GLP-1RAs during HSCT, the medications should be considered in patients early during their leukemia treatment with cessation prior to the start of the conditioning regimen. This allows for weight reduction prior to HSCT and avoidance of potential drug-drug interactions between GLP-1RAs and medications such as those used for graft versus host disease (GVHD) prophylaxis.

Moreover, in addition to improved glycemic control and weight loss, GLP-1RAs have been linked to immune system modulation and a cancer risk reduction in obese individuals (23). In fact, recent data indicates that the presence of GLP-1 contributes to increased inflammation following HSCT in pediatric patients, and the reduction of GLP-1 led to the reduction of inflammatory markers (24). This resulted in a reduced risk of acute GVHD and a decrease in markers associated with endothelial injury. While GLP-1RAs mimic the action of GLP-1, potentially leading to improved inflammation and weight loss without increasing GLP-1 production, the impact of GLP-1 receptor activation via GLP-1RAs on activating mechanisms of inflammation and endothelial injury is unclear. Pre-clinical data indicates that the activation of GLP-1R signaling using semaglutide did not significantly reduce gut injury and inflammation secondary to gut GVHD in mice (25). This further supports that the use of GLP-1RAs should be utilized early in the leukemic treatment phase to reduce downstream effects of obesity rather than during HSCT. Due to conflicting clinical and pre-clinical impact of GLP-1RAs in the peri-transplant period, these agents warrant further investigation in AYAs who are obese and require HSCT to better understand the rate of weight loss, feasibility, safety, drug-drug interactions, and long-term benefits.

#### Minimally invasive options

Along with pharmacological modulation, faster-acting, minimally invasive options to reduce BMI include intragastric balloons and endoscopic gastric plication. The FDA has approved balloons for patients ≥18 years, and studies support their use in pediatric and adolescent patients (26–29). Reported weight loss includes 16% total and up to 56% excess weight (27). Plication offers another option with data reporting near 14% reduction in 6 months (27, 30). Because weight loss during treatment can contribute to muscle loss and malnutrition, early involvement of a nutritionist and physical therapist would be essential.

Ghasoub et al.’s review of the impact of obesity and surgery on pharmacokinetics of certain drugs necessary for the remission of specific leukemias highlighted the importance of surgical timing and the impact that a gastric surgery can have on drug absorption (2). Their analysis included more invasive surgeries, such as gastric bypass, and less invasive procedures, such as gastric banding; both procedures demonstrated a negative effect on drug absorption, as evidenced by subtherapeutic drug levels. While the pharmacokinetic mechanisms of the drugs studied in Ghasoub et al.’s CML study are fundamentally different from those utilized for the treatment of acute leukemia, caution will be necessary with implementation of any intervention potentially altering the digestive tract’s influence on drug absorption. Obesity may impact treatment efficacy and supportive care in the peri and post-HSCT period – this includes the use of GVHD prophylaxis medications and of microbials. Thus, we suggest future studies evaluate these procedures after patients achieve deep molecular response or minimal residual disease and prior to the start of conditioning chemotherapy. Clinical trials studying these minimally invasive procedures would be required to ascertain timing in terms of the patient’s relative state of immunosuppression, risk of compromise of post-surgical wound healing, and time to HSCT. Nevertheless, the authors’ message is not to contraindicate surgical options in patients with leukemia but rather support the utilization of therapeutic drug monitoring and further study of treatment optimization in patients with obesity or gastrointestinal alterations.

#### Safety considerations

While GLP-1Ras are generally considered safe in otherwise healthy populations and have received approval for the treatment of adolescents with obesity, we acknowledge safety data is lacking for their use in patients undergoing chemotherapy, conditioning regimens and HSCT, or during periods of immunosuppression. Similarly, the safety of minimally invasive bariatric procedures following intensive chemotherapy has not yet been systematically studied. Several critical questions remain unaddressed: potential pharmacologic interactions between GLP-1RAs and chemotherapeutic agents, aspiration risk during periods of intensive conditioning, the effect of delayed gastric emptying on the risk and severity of gastrointestinal mucositis or GVHD, and the immunologic consequences of rapid weight loss in patients with profound bone marrow depletion. In a population at risk for malnutrition and sarcopenia secondary to disease, metabolic demands, and poor nutritional state, the impact of intentional weight loss on aspects such as muscle mass and nutrition absorption should not be overlooked. Careful monitoring and potential supplementation of macro- and micronutrients will need to be considered throughout study and treatment.

These uncertainties should not conclusively deter future implementation of GLP-1RAs or minimally invasive bariatric procedures in this patient population, but rather underscore the urgent need for focused investigation before such interventions can be definitively recommended in the context of HSCT, even as they represent potentially valuable strategies to improve transplant outcomes in AYA patients with obesity.

Health education to providers and patients will be paramount to support adequate drug dosing, nutritional balance, and ensure potential side effects are addressed should related acute and delayed effects of GLP-1RAs or surgery occur. However, in patients for whom previous weight loss strategies have not provided results necessary to place them in an optimal BMI stratum prior to HSCT, GLP-1RAs or minimally invasive procedures could provide a path to their curative treatment, despite gut alteration and subsequent need for medication dosing adjustments.

### Optimizing HSCT outcomes in AYAs through obesity management

The negative impact of obesity on outcomes following HSCT in AYA leukemia patients supports weight control prior to the initiation of the conditioning regimen leading to transplantation. As obesity rates rise, novel strategies are needed to reduce associated treatment risks. The need for a curative and safe HSCT additionally raises ethical concerns: delaying or denying access to experimental weight loss interventions if available, such as bariatric or pharmacologic therapies, risks compromising survival. Equitable, timely access to interventions such as we present here should be considered to support informed, timely, and life-saving treatment decisions.

Additional studies are needed to advance the works of Doney et al. and Ghasoub et al. to further support their findings and build stronger correlation between obesity and NRM in AYA patients undergoing HSCT. This will assist in further investigating the relationship between the impact of higher BMI and obesity and effectiveness of conditioning chemotherapy dosing, GVHD prophylaxis, and complications such as endotheliopathies and infections. Most importantly – literature is needed to address avenues for safe weight loss in this unique patient population that does not significantly impact drug absorption and wound healing. The value of entering intensive chemotherapy regimens or undergoing transplant at a BMI that is neither under- or overweight, may allow for improved treatment adherence, tolerance, and notably, transplant-related morbidity, resulting in improved outcomes.

## Conclusion

AYAs with obesity experience significantly worse outcomes in both acute leukemia and following HSCT used as consolidative therapy for high-risk disease. This disparity highlights the urgent need for proactive obesity management strategies, including minimally invasive procedures, pharmacologic options, lifestyle interventions, and strong social support systems. We call for the development of guidelines and focused research on safe, effective, pre-transplant interventions to reduce obesity-related morbidity and mortality in AYA patients undergoing HSCT. Implementing such strategies may mark the beginning of improving access to transplant, treatment- and weight-related morbidity, survival outcomes, and promote more equitable, life-saving care for this vulnerable patient population.
